# Supplementary material for: Gutka consumption and dietary partialities explaining anemia in women of a coastal slum of Karachi, Pakistan: A mixed-method study
Source: PLoS One. 2022 Oct 31;17(10):e0276893. doi: 10.1371/journal.pone.0276893 (PMC9621450; doi:10.1371/journal.pone.0276893)
Supplement: S1 Checklist — (DOC) [file pone.0276893.s001.doc]

STROBE Statement—Checklist of items that should be included in reports of ***cross-sectional studies***

|  | Item No | Recommendation |  |  |  |
| --- | --- | --- | --- | --- | --- |
| **Title and abstract** | 1 | (*a*) Indicate the study’s design with a commonly used term in the title or the abstract |  | Page 1, Title | |
|  |  | (*b*) Provide in the abstract an informative and balanced summary of what was done and what was found |  | Page 2, Abstract | |
| Introduction | | |  |  |  |
| Background/rationale | 2 | Explain the scientific background and rationale for the investigation being reported |  | Page 3-4, Introduction (line 70-110) | |
| Objectives | 3 | State specific objectives, including any prespecified hypotheses |  | Page 4, Introduction (line 110-113) | |
| Methods | | |  |  |  |
| Study design | 4 | Present key elements of study design early in the paper |  | Page 4, Methods under study design and study site | |
| Setting | 5 | Describe the setting, locations, and relevant dates, including periods of recruitment, exposure, follow-up, and data collection |  | Page 4, Methods under study design and study site and Page 5 under Data collection | |
| Participants | 6 | (*a*) Give the eligibility criteria, and the sources and methods of selection of participants |  | Page 5, Methods Sampling technique and eligibility | |
| Variables | 7 | Clearly define all outcomes, exposures, predictors, potential confounders, and effect modifiers. Give diagnostic criteria, if applicable |  | Page 5, Methods under Data collection tools and procedures and Page 6 under Data analysis | |
| Data sources/ measurement | 8* | For each variable of interest, give sources of data and details of methods of assessment (measurement). Describe comparability of assessment methods if there is more than one group |  | Page 5, Methods under Data collection tools and Data collection | |
| Bias | 9 | Describe any efforts to address potential sources of bias |  | Page 5, Methods under data collection (line 165) | |
| Study size | 10 | Explain how the study size was arrived at |  | Page 4, Methods under sample size | |
| Quantitative variables | 11 | Explain how quantitative variables were handled in the analyses. If applicable, describe which groupings were chosen and why |  | Page 6, Methods under Data Analysis | |
| Statistical methods | 12 | (*a*) Describe all statistical methods, including those used to control for confounding |  | Page67, Methods under Data Analysis | |
|  |  | (*b*) Describe any methods used to examine subgroups and interactions |  | Page 6, Methods under Data Analysis | |
|  |  | (*c*) Explain how missing data were addressed |  | Page 5, Methods under data collection (line 166) | |
|  |  | (*d*) If applicable, describe analytical methods taking account of sampling strategy |  | Page 4, Method under Sampling technique and eligibility. | |
|  |  | (*e*) Describe any sensitivity analyses |  | N/A | |
| Results | | |  |  |  |
| Participants | 13* | (a) Report numbers of individuals at each stage of study—eg numbers potentially eligible, examined for eligibility, confirmed eligible, included in the study, completing follow-up, and analysed |  | Page 6, Results and Figure 1 | |
|  |  | (b) Give reasons for non-participation at each stage |  | Page 6, Results and Figure 1 | |
|  |  | (c) Consider use of a flow diagram |  | Figure 1 | |
| Descriptive data | 14* | (a) Give characteristics of study participants (eg demographic, clinical, social) and information on exposures and potential confounders |  | Table 1 | |
|  |  | (b) Indicate number of participants with missing data for each variable of interest |  | Table 1, 2 and 3 | |
| Outcome data | 15* | Report numbers of outcome events or summary measures |  | Page 6, Results and Table 2-3 | |
| Main results | 16 | (*a*) Give unadjusted estimates and, if applicable, confounder-adjusted estimates and their precision (eg, 95% confidence interval). Make clear which confounders were adjusted for and why they were included |  | Page 6, Results and Table 2-3 | |
|  |  | (*b*) Report category boundaries when continuous variables were categorized |  | Page 6, Results and Table 2-3 | |
|  |  | (*c*) If relevant, consider translating estimates of relative risk into absolute risk for a meaningful time period |  | N/A |  |
| Other analyses | 17 | Report other analyses done—eg analyses of subgroups and interactions, and sensitivity analyses |  | N/A |  |
| Discussion | | |  |  |  |
| Key results | 18 | Summarise key results with reference to study objectives |  | Page 10, Discussion | |
| Limitations | 19 | Discuss limitations of the study, taking into account sources of potential bias or imprecision. Discuss both direction and magnitude of any potential bias |  | Page 10, Discussion | |
| Interpretation | 20 | Give a cautious overall interpretation of results considering objectives, limitations, multiplicity of analyses, results from similar studies, and other relevant evidence |  | Page 10, Discussion | |
| Generalisability | 21 | Discuss the generalisability (external validity) of the study results |  | Page 10, Discussion | |
| Other information | | |  |  |  |
| Funding | 22 | Give the source of funding and the role of the funders for the present study and, if applicable, for the original study on which the present article is based |  | Page 11, Declaration under funding | |

*Give information separately for exposed and unexposed groups.

**Note:** An Explanation and Elaboration article discusses each checklist item and gives methodological background and published examples of transparent reporting. The STROBE checklist is best used in conjunction with this article (freely available on the Web sites of PLoS Medicine at http://www.plosmedicine.org/, Annals of Internal Medicine at http://www.annals.org/, and Epidemiology at http://www.epidem.com/). Information on the STROBE Initiative is available at www.strobe-statement.org.
