# Supplementary material for: Gutka consumption and dietary partialities explaining anemia in women of a coastal slum of Karachi, Pakistan: A mixed-method study
Source: PLoS One. 2022 Oct 31;17(10):e0276893. doi: 10.1371/journal.pone.0276893 (PMC9621450; doi:10.1371/journal.pone.0276893)
Supplement: Attachment — Submitted filename: point-by-point response.docx [file pone.0276893.s003.docx]

**PONE-D-22-06122**

**Gutka Consumption and Dietary Partialities Explaining Anemia in Women of a Coastal Slum of Karachi, Pakistan: A Mixed-method Study**

**Point-by-point response**

| **Comments/Feedback** | | **Authors response** |
| --- | --- | --- |
| **Journal Requirements** | | |
| 1. | Please ensure that your manuscript meets PLOS ONE's style requirements, including those for file naming. | Thanks for highlighting this, we have now formatted the manuscript. |
| 2. | We note that the grant information you provided in the ‘Funding Information’ and ‘Financial Disclosure’ sections do not match. When you resubmit, please ensure that you provide the correct grant numbers for the awards you received for your study in the ‘Funding Information’ section. | Thanks for highlighting this, we have corrected this information. |
| 3. | Thank you for stating the following financial disclosure: The author(s) received no specific funding for this work. However, the author received support from the VITAL Pakistan Trust through existing maternal, neonatal and child health programs to conduct laboratory assessments. Please state what role the funders took in the study.  If the funders had no role, please state: "The funders had no role in study design, data collection and analysis, decision to publish, or preparation of the manuscript."  If this statement is not correct you must amend it as needed. Please include this amended Role of Funder statement in your cover letter; we will change the online submission form on your behalf. | Thanks for highlighting this, we have corrected this information.  Page 17: Declarations, funding |
| 4. | We note that you have indicated that data from this study are available upon request. PLOS only allows data to be available upon request if there are legal or ethical restrictions on sharing data publicly. For more information on unacceptable data access restrictions, please see http://journals.plos.org/plosone/s/data-availability#loc-unacceptable-data-access-restrictions.  In your revised cover letter, please address the following prompts:  a) If there are ethical or legal restrictions on sharing a de-identified data set, please explain them in detail (e.g., data contain potentially sensitive information, data are owned by a third-party organization, etc.) and who has imposed them (e.g., an ethics committee). Please also provide contact information for a data access committee, ethics committee, or other institutional body to which data requests may be sent.  b) If there are no restrictions, please upload the minimal anonymized data set necessary to replicate your study findings as either Supporting Information files or to a stable, public repository and provide us with the relevant URLs, DOIs, or accession numbers. For a list of acceptable repositories, please see http://journals.plos.org/plosone/s/data-availability#loc-recommended-repositories.  We will update your Data Availability statement on your behalf to reflect the information you provide. | We have updated this information in the manuscripts. We sought some guidance from previously published manuscripts in the PONE.  Page 17: Declarations, Data Availability Statement |
| 5. | We note that you have stated that you will provide repository information for your data at acceptance. Should your manuscript be accepted for publication, we will hold it until you provide the relevant accession numbers or DOIs necessary to access your data. If you wish to make changes to your Data Availability statement, please describe these changes in your cover letter and we will update your Data Availability statement to reflect the information you provide. | All relevant data is available within manuscript and based on reviewer comments, we have also added some supplementary information. We have made the changes in the data availability statement.  Page 17: Declarations, Data Availability Statement |
| 6. | Your ethics statement should only appear in the Methods section of your manuscript. If your ethics statement is written in any section besides the Methods, please move it to the Methods section and delete it from any other section. Please ensure that your ethics statement is included in your manuscript, as the ethics statement entered into the online submission form will not be published alongside your manuscript. | Thanks. We have moved this to methods sections now  Page 6: Ethics considerations |
| 7. | Please include your tables as part of your main manuscript and remove the individual files. Please note that supplementary tables (should remain/ be uploaded) as separate "supporting information" files | Thanks. This is done. |
| **Reviewer #1** | | |
| 8. | Placing the objectives against existing literature:  It will be useful to sharpen the introduction in a way that focuses on a) what we know about anemia in Pakistan,  b) what we know about nutritional outcomes of coastal communities and  c) what we know about the relationship between Gutka consumption and health outcomes. The last point in particular is lacking at the moment. The assumption appears to be that Gutka consumption reduces hunger, which in turn results in an inadequate food intake and thus malnutrition including anemia. However, what are the nutritional/ health implications of gutka consumption, what do we know about that from existing literature? | Thanks for the guidance on this. This helped us a lot to revisit the introduction and make it more distinct. Please check the changes under the section of Introduction, page 4, line 100 to 128 |
| 9. | Construction of key variables The study collected an impressive amount of primary data and yet a lot of that gets lost in the brief description of the research tools. I would suggest adding in a section that describes how key variables were constructed. For example, how was the 4- week food frequency score calculated and used? What are the different levels of food insecurity in the HFIAS? What was the premise behind picking seafood consumption as an indicator of a monotonous diet? Is seafood lacking in iron/ bioavailable iron? Was red meat consumption derived from the food frequency questionnaire or asked separately? How was Gutka consumption measured? The study is impressive in its collection of biomarkers for anemia. I would like to read more about the protocol that was followed for collection, storage and transportation of blood samples. What was the refusal rate specifically for the blood draws? A brief discussion of the limitations of using hemoglobin as an indicator of anemia. | Thank a lot for you appreciation and comments. We added detail about construction of variables and transformation in analysis with all the references.  Page 6, line 169-208  Specific to selection of seafood as a variable of interest in also added. Page 7, line 192-196  Further, we have added the refusal rate for blood draw. Also, during screening and eligibility those who refused for blood draw, were excluded as mentioned in the Figure 1. Only those were enrolled those who consented for both the study procedure i.e., survey and blood draws  Page 10, line 277  We have also added discussion of limitation and strength of using hemoglobin as an indicator of anemia. Page 16, line 465-471 |
| 10. | The role of the market versus own- production There is a lot of emphasis given to the role of red meat consumption for alleviating anemia in this community. It appears that consumption is low because of a preference for other foods as well as because of a high price of meat products. There is a lot of literature that has looked at the role of markets vs own- production for improved nutritional outcomes. More recently attention has focused on the increasing unaffordability of health diets. I am sharing some references below that might be useful, particularly for South Asia:  Gupta et al (2019). Food Policy. Women’s empowerment and nutrition status: the case of iron deficiency in India. Available at <https://www.sciencedirect.com/science/article/pii/S0306919219305858>  FAO State of Food security and nutrition in the world (2020) - Transforming Food Systems for Affordable Healthy Diets. Available at <https://www.fao.org/publications/sofi/2020/en/>  Gupta et al (2021). Global Food Security. Ground truthing the cost of achieving the EAT lancet recommended diets: Evidence from rural India. Available at <https://www.sciencedirect.com/science/article/pii/S2211912421000080> | Thank you so much to share this important expert opinion. This are very useful to shape our recommendations now. Page 17, line 488-502 |
| 11. | Policy implications  What are the policy implications of your results? What can be done to enable such communities to have access to healthy, diverse and affordable foods? Does it come from their own production, or market reforms, or alternate strategies like fortification/ supplementation possibly? | Thanks, this and point above were super helpful. Please check our recommendations now.  Page 17, line 488-502 |
| **Reviewer #2** | | |
| 12. | There are no tables in the manuscript!! I cannot comment on your results without looking at the tables. | Apologies for this. We have included all figures and tables at the end of manuscript now. |
| 13. | I agreed to review this paper because the proposed correlation between Gutka consumption and Anemia seemed interesting, however, there is no discussion on the reason why these two should be correlated. Why should we expect these two to be correlated? this discussion is crucial for this paper and currently it is entirely missing. | Thanks for mentioning this. We have added this information in the manuscript now.  Page 4, line 119-122, Page 15, line 421-430 |
| 14. | It is customary to present the organization of the paper at the end of the introduction. | Page 4, line 126 |
| 15. | In sample size, it is mentioned that sample size was increased to 557 considering dropouts and non-response. However, later in the paper, it is explained that total 600 women were considered, and final sample of eligible women was 557. In the Sample size section, 557 should be explained better. Currently, it is confusing. | Thanks for asking this. We have tried to clearly state that. Dropout of 8.4% was considered to apply attrition rate based on the assumption that some participant may withdraw from survey and/or blood draw after informed consent is given. Therefore, sample size was inflated to 557.  However, to reach the sample size of 557, team has approached to the household where MWRA were residing. Therefore, they to approached total 613 MWRA, 600 agreed for initial screening, 589 were eligible, 557 of them finally agreed and gave consent and enrolled. Figure 1. |
| 16. | Data collection was done in April and May. Authors should explain if hot weather or seasonal issues can affect the results. | Thanks for mentioning this. We have added this point in the strength and limitations, page 16, line 478 |
| 17. | What is the nutritional value of Gutka? | There is no scientific data available on this. However, based on what we know about ingredients, it has no nutrition value, or none reported. Page 16, line 454 |
| 18. | Do they consume Gutka because it is addictive or because they want to suppress hunger? If they eat their own catch and sea food is cheap, why do they need to suppress hunger? | Thanks, we have added this information. Page 15, line 424-437 |
| 19. | Why don’t they have poultry at home? it is usually a source of eggs in rural/slum households. | Thanks, we have added this information. Page 15, line 424-437 |
| 20. | How is the food insecurity measured? How is poverty or extreme poverty measured? it is mentioned in the discussion section. | We have added in information in data analysis, Page 7, Line 246 |
| 21. | How did you define balanced diet and were respondents made aware of this definition? | We did not use the term balanced diet with the participants, because it is difficult to communicate at community level. However, during our dissemination session with community, we used the term healthy and variety dietary option which community can access. , containing all key elements of nutrients. We have also changed this is the body of manuscripts where necessary. |
| 22. | In conclusion section, some recommendations for utility stores are mentioned. Since your discussion has been mostly around meat etc., do Utility Stores sell meat? | Thanks for sharing this, we have added this. Page 17, line 489 |
